# Identification of circulating sphingosine kinase-related metabolites for prediction of type 2 diabetes

**DOI:** 10.1186/s12967-021-03066-z

**Published:** 2021-09-16

**Authors:** Qi Chen, Wei Wang, Ming-Feng Xia, You-li Lu, Hua Bian, Chen Yu, Xiao-Ying Li, Mathew A. Vadas, Xin Gao, Huan-Dong Lin, Pu Xia

**Affiliations:** 1 Department of Endocrinology and Metabolism, Fudan Institute for Metabolic Diseases, Zhongshan Hospital, Fudan University, 180 Fenglin Road, Shanghai, 200032 China; 2 Central Laboratory, Xuhui Central Hospital, Shanghai, China; 3 Centenary Institute, The University of Sydney, Sydney, Australia

## Abstract

**Background:**

Sphingosine Kinase (SphK) that catalyzes sphingosine (Sph) to sphingosine 1-phosphate (S1P), plays a key role in both sphingolipid metabolism and cellular signaling. While SphK has been implicated in type 2 diabetes mellitus (T2DM), it is unexplored in humans. Herein, we investigated whether circulating SphK-related metabolites are associated with T2DM incidence in an established prospective cohort.

**Methods:**

Levels of SphK-related sphingolipid metabolites, including Sph, S1P, dihydrosphingosine (dhSph) and dihydro-S1P (dhS1P) in serum were measured by targeted-lipidomic analyses. By accessing to an established prospective cohort that involves a total of 2486 non-diabetic adults at baseline, 100 subjects who developed T2DM after a mean follow-up of 4.2-years, along with 100 control subjects matched strictly with age, sex, BMI and fasting glucose, were randomly enrolled for the present study.

**Results:**

Comparison with the control group, medians of serum dhS1P and dhS1P/dhSph ratio at baseline were elevated significantly prior to the onset of T2DM. Each SD increment of dhS1P and dhS1P/dhSph ratio was associated with 53.5% and 54.1% increased risk of incident diabetes, respectively. The predictive effect of circulating dhS1P and dhS1P/dhSph ratio on T2DM incidence was independent of conventional risk factors in multivariate regression models. Furthermore, combination of serum dhS1P and dhS1P/dhSph ratio with conventional clinical indices significantly improved the accuracy of T2DM prediction (AUROC, 0.726), especially for normoglycemic subjects (AUROC, 0.859).

**Conclusion:**

Circulating levels of dhS1P and dhS1P/dhSph ratio are strongly associated with increased risk of T2DM, and could serve as a useful biomarker for prediction of incident T2DM in normoglycemic populations.

## Introduction

Type 2 diabetes mellitus (T2DM) is a major global health problem that currently affects 463 million people over the world [1]. It is estimated that about 4.2 million deaths were attributable to diabetes in 20–79 years old adults, which accounted for 11.3% of total deaths globally in 2019 [2]. The most effective way to reduce such a global health burden is to prevent diabetes itself, because prevention of incident diabetes could reduce the all-cause mortality and the death from cardiovascular disease by 38.8%, and 44.2%, respectively [3]. However, the current strategy of prevention is still insufficient to curb the escalating epidemics of T2DM [1]. It is widely believed that both energy-dense diet and sedentary lifestyle are the primary causes of the epidemics of T2DM, however a substantial rise of incident diabetes was also seen in people with normal body weight or from the countries that have no major changes in the category of food consumption in recent decades [4]. Therefore, new approaches are urgently needed for better prediction and prevention of diabetes in general populations.

Sphingolipids are a class of essential lipids that function as both cell membrane constituents and signaling messengers [5]. Sphingosine kinase (SphK) is a key enzyme in sphingolipid metabolic network, which catalyzes the conversion of ceramides/sphingosine (Sph) to sphingosine 1-phosphate (S1P). Because the upstream substrates and the products of this enzyme often exhibit opposing biological functions, SphK is regarded as a "switch" of the sphingolipid rheostat [6]. There are two isoforms of SphK in human, i.e., SphK1 and SphK2, encoded by two different genes [6, 7]. Both SphK1 and SphK2 catalyzes the conversion of Sph to S1P, while SphK2 can preferably catalyze the formation of dihydro-S1P (dhS1P) from dihydrosphingosine (dhSph) in mammals [7]. Besides, SphK1 and SphK2 often possess distinct tissue distribution, subcellular localization and biochemical properties, and thus exhibit different and even opposite functions in a context-dependent manner [7, 8]. For instance, we have recently reported that global deletion of *Sphk1* in mice resulted in a significant loss of pancreatic β-cell mass and promoted the onset of diabetes [9], whereas *Sphk2* deficiency ameliorated diabetic phenotype by protecting β-cells against lipoapoptosis [10]. These findings indicate that both SphK1 and SphK2 are critically involved in the regulation of β-cell viability and function in opposite directions. Moreover, the implications of SphK1 and SphK2 in insulin resistance have recently emerged. For examples, Anderson et al. reported that adipocyte-specific deletion of *Sphk1* in mice impaired glucose tolerance and elevated fasting blood glucose, which is believed attributable to the suppressed lipolysis in adipocytes [11]. More recently, we have reported that hepatocyte-specific deficiency of *SphK2* in mice increased hepatic glucose production, impaired glucose homeostasis and caused insulin resistance [12]. Thus, it appears that SphK is critically involved in both insulin resistance and β-cell failure, suggesting a crucial role in diabetes. However, the relationships of SphK and its related sphingolipid metabolites with diabetes in humans remain unexplored. Therefore, the present study aimed to investigate the association of SphK-related metabolites with the risk of T2DM in a community-based prospective cohort.

## Methods

### Study samples

For the animal study, the colonies of *Sphk1*^−/−^, *Sphk2*^−/−^ and wild-type littermates were derived on the same C57BL/6 background as detailed in our previous studies [9, 10]. All mice were housed in a temperature-controlled, pathogen-free environment on a 12-h light/dark cycle and had ad libitum access to food and water. Animal studies were approved by the Animal Use and Care Committee of Fudan University and conformed with the U.S. National Institutes of Health (NIH) U.S. Department of Health and Human Services Public Health Service Policy on Humane Care and Use of Laboratory Animals (NIH publication No. 15-8013; Bethesda, MD, USA).

For the cohort study, the participants were enrolled from the Shanghai Changfeng Study, a population-based prospective cohort study on metabolic diseases in Chinese community residents aged over 45 years as previously reported in details [13, 14]. A retrospective nested case–control study was conducted. Among 2486 non-diabetic participants at baseline, a total of 100 participants with newly diagnosis of T2DM after a mean follow-up of 4.2 years (3.5–5 years) were randomly selected and each case was matched with an individual without incident diabetes from the same cohort. All cases and control subjects were strictly matched with respects to age, sex, body mass index (BMI; ± 1 kg/m^2^) and fasting plasma glucose (FPG; ± 0.1 mmol/L) at baseline. All study participants provided written informed consent, and the study was approved by the Ethical Committee of Zhongshan Hospital affiliated to Fudan University (No. 2008-119).

### Anthropometric and biochemical profiling

Weight, height and waist circumference were measured by trained interviewers. BMI was calculated by dividing the weight (kg) by the square of the height (m^2^). The mean value of three resting blood pressure (BP) measurements was used for the analyses. Venous blood samples were collected for the biochemical examinations after an overnight fast of at least 12 h. Serum total cholesterol, high-density lipoprotein (HDL) cholesterol, triglycerides (TG) levels and uric acid were measured by an oxidase method. Low-density lipoprotein (LDL) cholesterol was calculated using the Friedewald equation. All participants underwent a 75 g oral glucose tolerance test (OGTT). Fasting and 2-h post-load plasma glucose (PPG) were measured using a glucose oxidase method. Serum insulin concentrations were measured using electrochemiluminescence immunoassay. Homeostatic Model Assessment of Insulin Resistance (HOMA-IR) was calculated by multiplying the FPG (mmol/L) by fasting insulin (μU/mL) and dividing by 22.5 [15]. For both the baseline and follow-up evaluations, diabetes was diagnosed if FPG ≥ 7.0 mmol/l or OGTT 2 h PPG ≥ 11.1 mmol/l, and normal glucose tolerance defined as FPG < 6.1 mmol/L and OGTT 2 h PPG < 7.8 mmol/L, according to the WHO criteria [16].

### Sphingolipid analyses by HPLC–MS/MS

For the measurement of Sph, dhSph, S1P and dhS1P, the serum samples collected from the study subjects were removed from − 80 °C storage just before the measurement. Sphingolipids were measured in duplicate of each sample (100 μL each), using a High Performance Liquid Chromatography-Tandem Mass Spectrometry (HPLC–MS/MS) methodology as previously described with some modifications[9, 17]. Briefly, the samples were mixed with 10 μL of internal standards (S1P-d7; Avanti, Alabama, USA) and 500 μL of extraction solution consisting of isopropanol, methanol and formic acid (45:45:10 v/v). Then, the mixtures were vortexed, sonicated and centrifuged at 1500×*g* for 10 min at 4 °C. Supernatants were diluted with 30% methanol before analysis. The samples were analyzed by positive-ion Ultrahigh-Performance Liquid Chromatography-Electrospray Ionization-Tandem Mass Spectrometry (UPLC-ESI–MS) in a multiple reaction monitoring mode performed on a Shimadzu LC-20AD liquid chromatography instrument (Shimadzu, Japan) and coupled to an QTrap5500 mass spectrometer (Applied Biosystems Sciex, Canada). Chromatographic separations were performed under a gradient elution on an Eclipse XDB-C8 (2.1 × 100 mm, 1.8 μm) column (Agilent Technologies) using a binary solvent system at a flow rate of 300 μl/min. Prior to sample injection, the column was equilibrated for 2 min with a solvent mixture of 70% mobile phase A (methanol/H2O/formic acid, 69/29/2/, v/v/v, with 2 mmol ammonium formate) and 30% phase B (methanol/tetrahydrofuran/formic acid, 97/2/1, v/v/v, with 2 mmol ammonium formate) as previously described[18]. After injection, the mobile phase A/B ratio was maintained at 70/30 for 2 min, followed by a linear gradient to 100% B over 5 min. The flow was then maintained for 10 min at 100% B, followed by a wash of the column with 70:30 A/B for 0.5 min before the next run. The mass transitions were m/z 300.2 → 282.2 for Sph, 302.2 → 254.2 for dhSph, 380.2 → 264.2 for S1P, 382.2 → 266.2 for dhS1P, 387.3 → 271.3 for S1P-d7, respectively. Levels of Sph, dhSph, S1P and dhS1P were quantified using external standard curves ranging from 0.1 to 1000 nmol, and normalized to each the synthetic internal standard (Avanti, Alabama, USA). All of the data were acquired and processed using Analyst 1.6 software from Sciex Inc., Concord, ON, Canada.

### Statistical analysis

The estimation of sample size was based on our preliminary experiment in 30 pairs of subjects. According to the preliminary data, subjects with incident diabetes had significantly higher log_10_ dhS1P/dhSph ratio levels of 0.04 with a SD of 0.11. Therefore, we determined that a sample size of 82 achieves 90% power to detect a mean of paired differences of 0.04 with an estimated standard deviation of differences of 0.11 and a significance level (alpha) of 0.05 using PASS tests for paired means. Assuming a drop-out rate of 20%, approximately 100 pairs subjects would be required.

All statistical analyses were performed using STATISTICAL ANALYSIS SYSTEM (SAS) 9.2 and R 3.4.2 software (SAS Institute). The continuous data were compared using the Student’s t-test, whereas the χ2 test was used for comparisons of categorical variables. Skewed variables were presented as the median with the interquartile range. The sphingolipids were non-normally distributed, so the sphingolipid parameters were log-transformed before analysis. Since the Shanghai Changfeng Cohort Study is a fixed cohort study with an average of 4.2-year (3.5–5 years) follow-up, multivariate logistic regression models were used to estimate the odds ratios (ORs) and 95%CIs of incident diabetes per-SD increase of each individual sphingolipid parameter at baseline after multiple adjustment. Model 1 was adjusted for age, sex and BMI changes as the basic model. Model 2 was adjusted for family history of diabetes, FPG and OGTT 2 h PPG in addition to factors included in Model 1. Model 3 was adjusted for serum total cholesterol, triglycerides, systolic blood pressure and uric acid in addition to factors included in Model 2. The performance of each serum sphingolipid component at baseline in prediction of incident diabetes was evaluated using receiver operating characteristic (ROC) curve analyses, and was compared with conventional risk factors of diabetes. A combination model of serum sphingolipids with conventional risk factors was also established and assessed. Subgroup analysis was performed in the participants with normal glucose tolerance. All tests were two-sided and a *P* value of less than 0.05 was considered statistically significant.

## Results

### Circulating levels of the SphK-related sphingolipid metabolites in mice

**Fig. 1:**
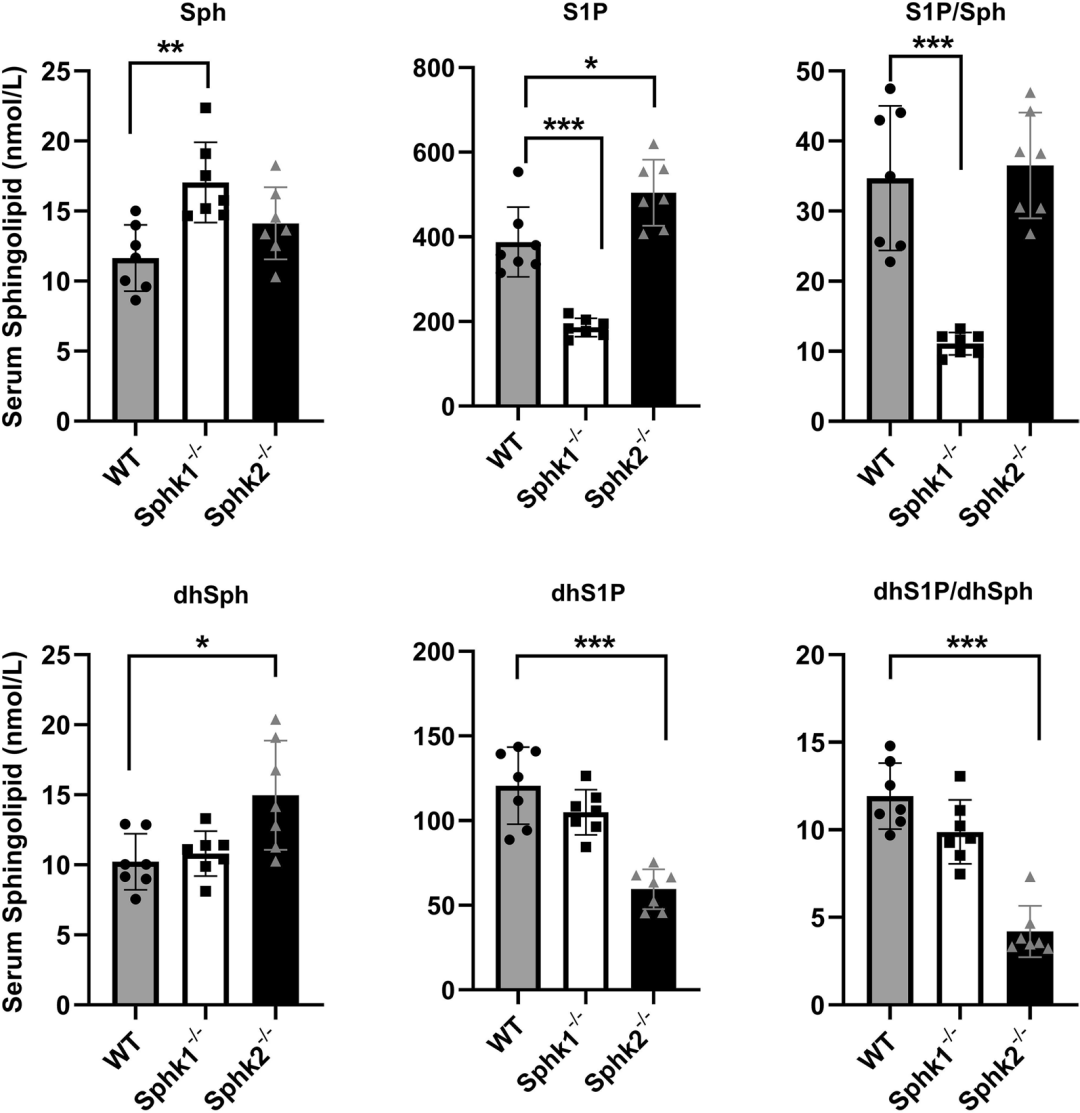
Circulating levels of SphK-related sphingolipids in *Sphk1*^*−/−*^ and *Sphk2*^*−/−*^ mice. Levels of circulating SphK-related sphingolipids, including sphingosine (Sph), sphingosine 1-phosphate (S1P), dihydro-Sph (dhSph) and dhS1P were measured in *Sphk1*^*−/−*^, *Sphk2*^*−/−*^ and wild-type (WT) mice (n = 7 per group). Ratios of S1P/Sph and dhS1P/dhSph are shown in the right panels. Data are expressed as means ± SD. *P < 0.05, **P < 0.01, ***P < 0.001

Because no established methodology currently available for measuring SphK activity in vivo, we applied *Sphk1*-deficient (*Sphk1*^−/−^) and *Sphk2*-deficient (*Sphk2*^−/−^) mice to measure circulating levels of the key sphingolipid metabolites that are mostly relevant to SphK activity, including Sph, dhSph, S1P and dhS1P, using HPLC–MS/MS-based targeted-lipidomic analyses. In line with the enzymatic property of SphK, i.e., converting Sph to S1P, *Sphk1*^−/−^ mice resulted in a significant decrease in serum S1P but increase in Sph levels (Fig. 1). Correspondingly, the ratio of circulating S1P/Sph was dramatically decreased. However, the S1P/Sph ratio was not significantly altered in *Sphk2*^−/−^ mice, as S1P levels were significantly increased in *Sphk2*^−/−^ mice. The latter was consistent with the previous reports[19, 20]. By contrast, *Sphk2*^−/−^ significantly decreased dhS1P and slightly increased dhSph levels, and thus a remarkable reduction in serum dhS1P/dhSph ratio was detected. However, the circulating dhS1P/dhSph ratio was not significant altered by *Sphk1*^−/−^, although a decrease trend existed (Fig. 1). Taken together, these results indicate that the in vivo activity of SphK1 and SphK2 is likely reflected in the circulating ratio of S1P/Sph and dhS1P/dhSph, respectively.

### Circulating levels of the SphK-related sphingolipid metabolites in human

**Fig. 2:**
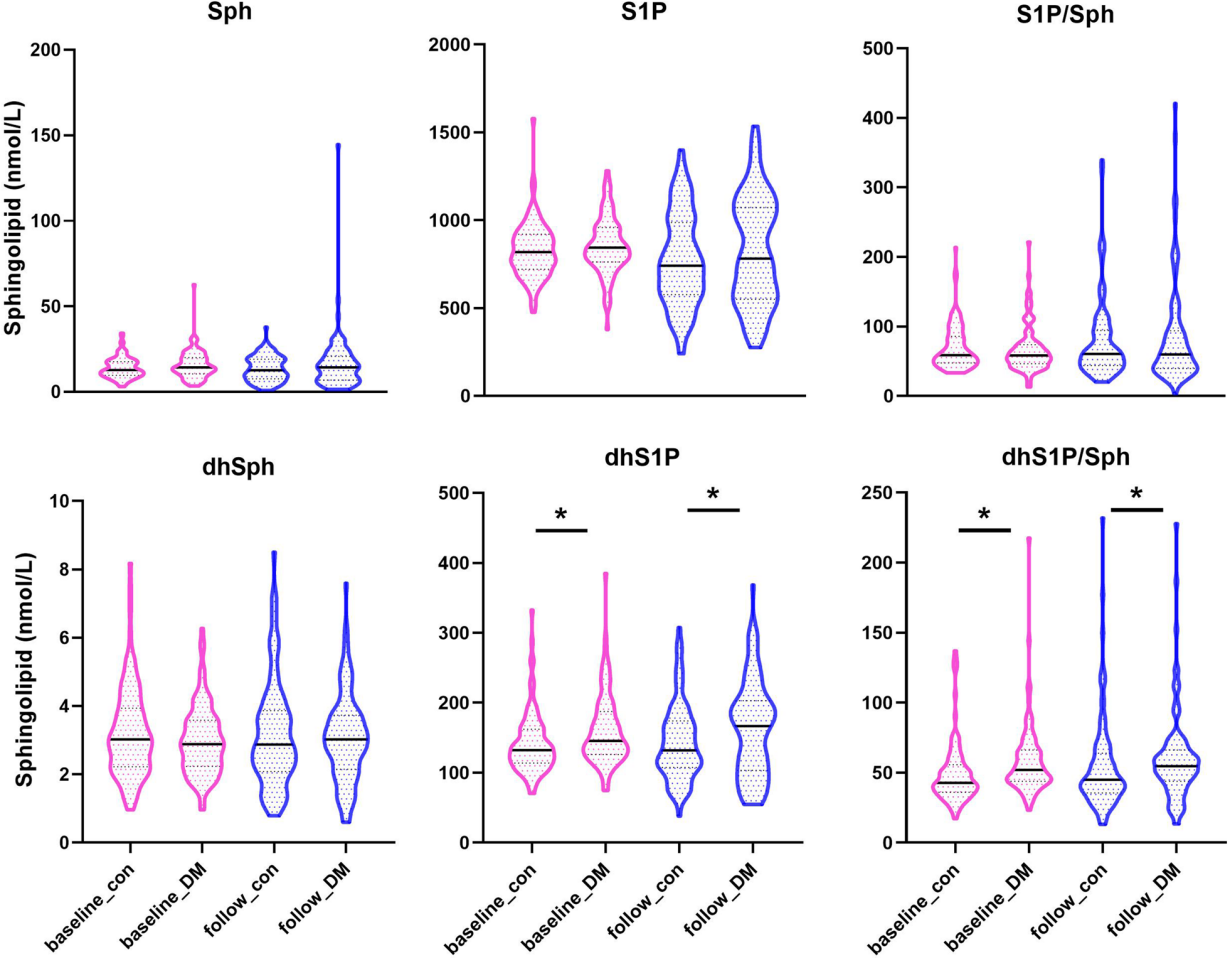
Circulating dhS1P and dhS1P/dhSph ratio are elevated before the occurance of T2DM. Levels of circulating SphK-related sphingolipids, including Sph, S1P, dhSph and dhS1P were measured in the control (n = 100) and incident diabetic groups (n = 100) at the time points of baseline and follow-up, respectively. Data are expressed as median with the interquartile range. **P < 0.01, incident diabetes compared with the controls

We have recently reported that global deletion of either *Sphk1* or *Sphk2* exhibited a significant impact on diabetes in the animal models, i.e., loss of *Sphk1* promotes, but *Sphk2* deficiency protects, the disease [9, 10]. We thus attempted to apply these experimental findings into a clinically relevant setting and investigate whether SphK is associated with T2DM incidence in humans. To this end, we conducted targeted-lipidomic analyses of the SphK-related sphingolipids in serum collected from an established community-based prospective cohort with a nested case–control design. We randomly selected 100 cases who developed T2DM (incident T2DM) after a mean follow-up of 4.2 years, which were matched with 100 subjects who did not develop diabetes (serving as a control group) from a total of 2486 participants without diabetes at baseline in the cohort. The two groups were strictly matched in subjects' age, sex, baseline BMI and FPG levels. Baseline characteristics of the study population are shown in Table 1. In addition to the matched parameters, there were no difference in waist circumference, blood pressure, serum LDL-C, HDL-C, uric acid, 2-h PPG and HOMA-IR between the two groups. However, levels of total cholesterol in the incident diabetic group were higher than the control group (5.32 (4.70–5.89) vs 4.89 (4.44–5.51), *P* = 0.010) at baseline. Remarkably, compared with the control group, the subjects with incident diabetes had significantly higher levels of baseline dhS1P (132.0 nmol/L vs 153.0 nmol/L, *P* = 0.002) and dhS1P/dhSph ratio (42.2 nmol/L vs 50.7 nmol/L, *P* < 0.001). We then compared the general characteristics and SphK-related sphingolipids after the follow-up. The significant differences in dhS1P (132.2 nmol/L vs 166.6 nmol/L, *P* = 0.021) and dhS1P/dhSph ratio (45.0 nmol/L vs 54.8 nmol/L, *P* = 0.024) remained between the two groups at the end of follow-up (Table 2 and Fig. 2). However, there were no significant difference in serum levels of Sph, S1P and dhSph and ratio of S1P/Sph between the control and incident diabetic group either at baseline or after follow-up.

**Table 1:** Baseline characteristics and levels of SphK-related sphingolipids in the control and incident T2DM groups

Characteristic	Control (n = 100)	Incident T2DM (n = 100)	*P* value
Men, n (%)	41 (41%)	41 (41%)	0.999
Age, years	62.4 (56.8–70.0)	62.2 (56.1–67.6)	0.729
Body mass index, kg/m^2^	24.7 ± 2.5	24.5 ± 2.8	0.744
Waist circumference, cm	85.1 ± 8.5	84.8 ± 9.3	0.809
Total Cholesterol (mmol/L)	4.89 (4.44–5.51)	5.32 (4.70–5.89)	0.010
LDL (mmol/L)	2.86 ± 0.80	3.03 ± 0.73	0.121
HDL (mmol/L)	1.33 (1.20–1.61)	1.32 (1.17–1.51)	0.743
Triglycerides (mmol/L)	1.41 (1.07–1.96)	1.54 (1.15–2.23)	0.409
Fasting plasm glucose (mmol/L)	5.10 (4.85–5.30)	5.10 (4.90–5.50)	0.303
OGTT 2 h-plasm glucose (mmol/L)	7.30 (5.85–8.70)	7.40 (6.30–8.80)	0.213
Insulin (μU/mL)	8.00 (5.77–10.75)	8.30 (5.97–10.97)	0.777
HOMA-IR	1.90 (1.34–2.44)	1.92 (1.35–2.44)	0.714
Uric acid (μmol/L)	315.0 (265.0–356.0)	315.5 (267.0–373.8)	0.788
Family history of diabetes, n (%)	28 (28%)	27 (27%)	0.874
Systolic blood pressure (mmHg)	133.8 ± 15.5	138.5 ± 18.1	0.052
Diastolic blood pressure (mmHg)	77.1 ± 10.1	78.8 ± 10.0	0.231
Sphingolipids (nmol/L)			
Sph	12.7 (9.58–17.6)	14.2 (10.5–20.0)	0.116
S1P	818.0 (724.0–919.0)	844.0 (763.0–958.0)	0.151
S1P/Sph	58.2 (46.8–82.3)	55.2 (43.0–72.2)	0.489
dhSph	3.09 (2.25–4.18)	2.95 (2.29–3.63)	0.281
dhS1P	132.0 (109.5–163.3)	153.0 (128.0–189.2)	0.002
dhS1P/dhSph	42.2 (34.0–52.6)	50.7 (44.0–64.7)	< 0.001

Data are presented as mean ± SD, except for skewed variables, which are presented as the median with the interquartile range given in parentheses

HOMA-IR: homeostasis model assessment of insulin resistance

**Table 2:** General characteristics and levels of SphK-related sphingolipids at follow-up in the control and incident T2DM groups

Characteristic	Control (n = 100)	Incident T2DM (n = 100)	*P* value
Age, years	66.8 (60.3–74.0)	66.3 (60.3–72.2)	0.999
Body mass index, kg/m^2^	24.9 ± 3.1	25.7 ± 3.4	0.080
Waist circumference, cm	83.1 ± 9.2	85.5 ± 9.8	0.083
Total Cholesterol (mmol/L)	5.06 (4.41–5.64)	5.13 (4.37–5.80)	0.500
LDL (mmol/L)	2.94 ± 0.80	2.87 ± 0.89	0.523
HDL (mmol/L)	1.42 (1.12–1.69)	1.33 (1.16–1.62)	0.602
Triglycerides (mmol/L)	1.37 (1.06–1.78)	1.72 (1.27–2.30)	0.026
Fasting plasm glucose (mmol/L)	5.30 (5.10–5.60)	6.10 (5.45–7.25)	< 0.001
OGTT 2 h-plasm glucose (mmol/L)	6.10 (5.45–6.90)	12.1 (11.3–13.8)	< 0.001
Insulin (μU/mL)	6.90 (5.00–9.80)	10.8 (6.50–15.7)	< 0.001
HOMA-IR	1.62 (1.11–2.33)	2.81 (1.69–4.81)	< 0.001
Uric acid (μmol/L)	309.0 (256.0–379.5)	321.0 (265.5–377.5)	0.774
Systolic blood pressure (mmHg)	138.0 ± 20.2	142.0 ± 18.4	0.149
Diastolic blood pressure (mmHg)	77.1 ± 11.5	78.5 ± 8.6	0.335
Sphingolipids (nmol/L)			
Sph	12.6 (7.58–19.1)	14.3 (6.8–20.7)	0.110
S1P	740.5 (575.7–987.1)	781.1 (550.3–1072)	0.225
S1P/Sph	60.3 (43.8–94.1)	59.9 (39.6–93.8)	0.497
dhSph	2.88 (2.09–3.87)	3.03 (2.15–3.74)	0.570
dhS1P	132.2 (107.7–174.2)	166.6 (103.6–203.3)	0.021
dhS1P/dhSph	45.0 (35.6–63.9)	54.8 (44.0–66.3)	0.024

Data are presented as mean ± SD, except for skewed variables, which are presented as the median with the interquartile range given in parentheses

### Association of the SphK-related sphingolipids with incident T2DM

Having shown the elevated level of dhS1P and dhS1P/dhSph ratio occurred prior to diabetes onset in the case group, we then further evaluated the independent association of each sphingolipid component at baseline with the risk of incident diabetes by multiple logistic regression analyses. As shown in Table 3, univariate regression analysis revealed that levels of serum dhS1P and dhS1P/dhSph ratio, but not others, were significantly and positively associated with increased risk of incident diabetes, and each SD increment of dhS1P and dhS1P/dhSph ratio was associated with 53.5% (OR, 1.54; 95%CI, 1.12–2.10) and 54.1% (OR, 1.54; 95%CI, 1.10–2.17) increased risk of incident T2DM, respectively. Parameter estimates of the correlation between baseline dhS1P level and dhS1P/dhSph ratio and risk of incident diabetes remained significant after successively adjusted for age, sex, BMI changes, family history of diabetes, FPG, 2-h PPG, as well as the addition of total cholesterol, triglycerides, systolic blood pressure and uric acid levels (dhS1P: OR, 1.50; 95%CI, 1.05–2.14; dhS1P/dhSph: OR, 1.70; 95%CI, 1.14–2.53).

**Table 3:** Odds ratios (95%CI) for incident diabetes per SD increase in individual sphingolipid component

	per SD (nmol/L)	Unadjusted		Model 1		Model 2		Model 3	
		OR (95%CI)	*P* value	OR (95%CI)	*P* value	OR (95%CI)	*P* value	OR (95%CI)	*P* value
Sph	7.22	1.283 (0.953–1.729)	0.101	1.328 (0.967–1.823)	0.080	1.343 (0.974–1.852)	0.072	1.244 (0.878–1.764)	0.220
S1P	159.7	1.190 (0.896–1.579)	0.229	1.229 (0.911–1.657)	0.177	1.247 (0.918–1.696)	0.158	1.113 (0.801–1.545)	0.524
S1P/Sph	32.6	0.915 (0.692–1.210)	0.534	0.944 (0.705–1.265)	0.701	0.946 (0.705–1.269)	0.711	0.999 (0.732–1.364)	0.996
dhSph	1.18	0.808 (0.608–1.075)	0.144	0.804 (0.593–1.092)	0.163	0.818 (0.600–1.115)	0.204	0.711 (0.507–0.997)	0.048
dhS1P	48.0	1.535 (1.124–2.096)	0.007	1.586 (1.137–2.212)	0.007	1.632 (1.146–2.326)	0.007	1.498 (1.050–2.138)	0.026
dhS1P/dhSph	24.1	1.541 (1.095–2.168)	0.013	1.536 (1.084–2.176)	0.016	1.546 (1.065–2.244)	0.022	1.695 (1.137–2.526)	0.009

Data were odds ratio (95% confidence interval)

Model 1 included terms for age, sex and BMI changes

Model 2 included terms for age, sex, BMI changes, family history of diabetes, FPG and OGTT 2 h-blood glucose

Model 3 included terms for age, sex, BMI changes, family history of diabetes, FPG, OGTT 2 h-blood glucose, total cholesterol, triglycerides, systolic blood pressure and uric acid

### Performance of the SphK-related sphingolipids for prediction of incident T2DM

**Fig. 3:**
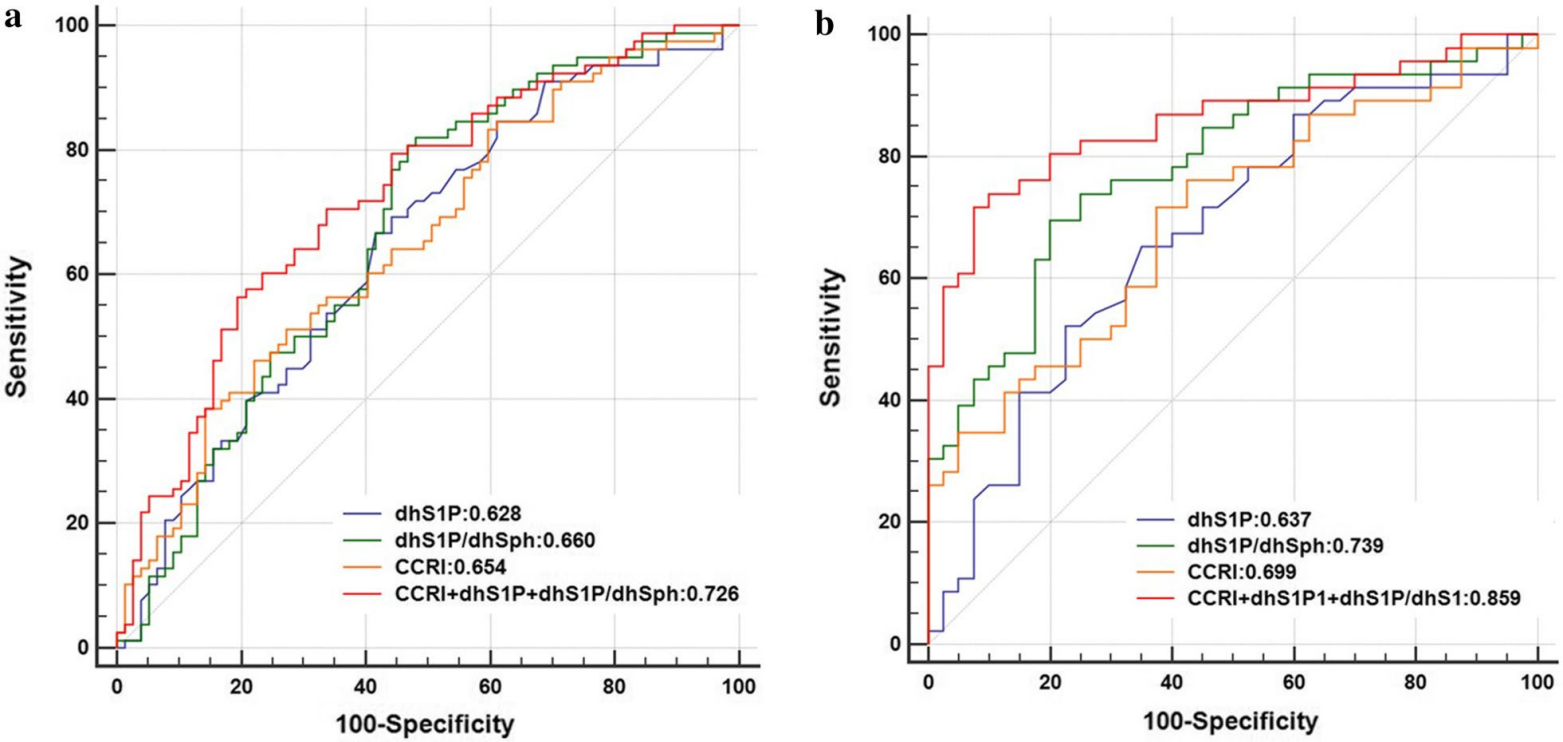
Relative contribution of dhS1P and dhS1P/dhSph ratio to prediction of T2DM. The performance of dhS1P and dhS1P/dhSph ratio in prediction of incident diabetes was evaluated by ROC curve analysis, and compared with the combined conventional risk indicators (CCRI) that include age, BMI, LDL, HDL, triglycerides, FPG, OGTT 2 h PPG, HOMA-IR and systolic blood pressure. Plots show area under the curves in **a** all study subjects and **b** the subjects with normal glucose tolerance

In attempt to assess the potential value of serum dhS1P and dhS1P/dhSph ratio at baseline for prediction of diabetes, we performed receiver-operating-characteristic curves (ROC) analyses. The data indicate that the prediction performance of serum dhS1P and dhS1P/dhSph ratio for the incidence of diabetes was comparable to that of the combined conventional risk models, including age, BMI, LDL, HDL, triglycerides, FPG, OGTT 2 h PPG, HOMA-IR and systolic blood pressure. A combination of serum dhS1P and dhS1P/dhSph with the conventional diabetes risk factors could further improve the accuracy for prediction of incident diabetes (AUROC, 0.726; 95%CI, 0.647–0.805), in comparison with the conventional prediction models (AUROC, 0.654; 95%CI, 0.568–0.739) (Fig. 3a). Furthermore, in subgroup analysis when dhS1P and dhS1P/dhSph ratio were added to established risk prediction model of T2DM (AUROC,0.699; 95%CI, 0.590–0.793) in subjects with normal glucose tolerance at baseline, the prediction was significantly improved to an AUROC of 0.859 (95%CI, 0.767–0.924) (*P* = 0.002 compared with conventional diabetes prediction models (Fig. 3b).

## Discussion

Early prediction of T2DM is regarded as one of the most effective ways to reduce its incidence in the general population and improve the long-term prognosis of the disease. The current study based on an established prospective cohort with a nested case–control design has for the first revealed time that elevated circulating levels of dhS1P and its ratio to dhSph were able to effectively predict diabetes incidence in otherwise healthy, normoglycemic individuals. The levels of dhS1P and dhS1P/dhSph ratio were indeed elevated before the changes of blood glucose and insulin levels in the subjects who developed T2DM after an average of 4.2-year follow-up, suggesting that these SphK-related sphingolipids could serve as a potential biomarker for early prediction of T2DM.

Previous human studies have reported that several sphingolipids, especially ceramides, were associated with insulin resistance (reviewed in [21, 22]). Based on a large family-based cohort study, the Strong Heart Family Study of American Indians, it was found that higher levels of serum ceramides with saturated fatty acids are associated with higher fasting serum insulin and HOMA-IR, both cross-sectionally and prospectively [23]. More recently, by using large-scale lipidomic analysis, studies on 2 ethnically distinct cohorts have shown that ceramides correlated positively whereas hexosylceramides correlated negatively with clinical and biochemical characteristics of obesity and diabetes [24, 25]. Notably, ceramides in serum are actually present in a broad variation of subspecies, which consist of either saturated or unsaturated sphingoid bases (i.e., dhSph and Sph) with different acyl-chain lengths of fatty acid (C14:0–C30:0) [26]. However, only a certain subspecies of ceramides can be found associated with insulin resistance in both experimental animals and humans [27, 28]. This strongly suggests the notion that the balance of sphingolipid metabolism, rather than ceramide accumulation, in blood is associated with insulin resistance and diabetes [24, 25, 27, 28]. While the regulation of circulating level of sphingolipids has yet been identified, recent studies have suggested that the liver is a major contributor of circulating ceramide species [29]. More recently, by taking the advantage of studies on hepatocyte-specific *Sphk2*-deficient mice, we found that the liver is also a key organ that regulates circulating levels of sphingolipids, including Sph, dhSph, S1P and dhS1P [12]. Remarkably, the liver-specific deficiency of *Sphk2* exhibits pronounced insulin resistance and glucose intolerance, demonstrating a key role of hepatic SphK2 in the regulation of insulin sensitivity and glucose homeostasis.

Similar to SphK1, SphK2 is also responsible for the conversion of Sph to S1P in cells. However, circulating S1P appears chiefly attributable to SphK1 activity [30, 31]. Indeed, we found that global deletion of *Sphk1* in mice results in a more than 50% reduction of S1P in serum, whereas loss of *Sphk2* unexpectedly increases circulating S1P (Fig. 1), which is consistent with previous reports [19, 20]. The circulating S1P is highly dynamic and its balance is regulated by a process that involves de-phosphorylation of S1P by S1P-phosphatase, re-phosphorylation of Sph by SphK2 and irreversible degradation of the newly formed S1P by its specific lyase [32]. Unlike SphK1, SphK2 can also specifically catalyze the phosphorylation of dhSph to form dhS1P in mammals [33]. Correspondingly, while deletion of *Sphk2* resulted in a significant decrease in dhS1P and increased dhSph in serum, neither dhS1P nor dhSph was influenced by *Sphk1* deficiency in mice (Fig. 1). In unstimulated cells, SphK1 is a cytosolic enzyme, whereas SphK2 can be found in the nucleus, the cytoplasm, and other intracellular compartments. Although it was reported that a minor fraction of SphK1 is constitutively exported from endothelial cells, possibly contributing to circulating S1P [34], we were unable to detect the enzymatic activity of either SphK1 or SphK2 in murine and human serum. Thus, the findings that circulating S1P/Sph and dhS1P/dhSph ratios were dramatically decreased by deficiency of *Sphk1* and *Sphk2*, respectively, indicate that the ratios could represent a favorable readout of in vivo activity for these two isoenzymes.

With nearly 4000 sphingolipid metabolites known to be present in mammalian cells (http://www.lipidmaps.org), many of which are bioactive and play important roles in biological function. Despite recent progress in mass spectrometry-based technologies that allow for better discrimination of individual sphingolipid species than was previously possible, only a relatively small portion of sphingolipids can be detected in blood by non-targeted sphingolipidomic or large-scale lipidomic analyses [35, 36]. The current study was driven primary by our previous experimental data that suggest an important role of SphK in the pathogenesis of diabetes. Thus, we aimed to explore the relationship between sphingolipids and T2DM with a focus on SphK-related metabolites, including Sph, dhSph, S1P and dhS1P. The most significance of the current study is the finding that high levels of circulating dhS1P and its ratio to dhSph are prospectively associated with T2DM incidence, revealing a novel biomarker for susceptibility to develop diabetes, up to 5 years before the disease diagnosed. The prediction performance of circulating dhS1P/dhSph ratio is better than dhS1P alone. In marked contrast, neither S1P levels nor S1P/Sph ratio is associated with the risk of incident diabetes. To the best of our knowledge, this is the first study showing the association of T2DM incidence with circulating levels of SphK-related sphingolipids in a prospective epidemiologic setting. Early identification of individuals with high risk of incident diabetes is of critical importance, because diabetes and its complications have been fully proven to be preventable [37, 38]. Although conventional risk factors, such as FPG, BMI and dyslipidemia, could predict incident diabetes to a certain degree [39], their prediction accuracy and sensitivity are limited. A better clinical prediction model for incident diabetes remains urgently needed. Previous efforts on combining the genetic risk factors of T2DM with conventional predictors add modestly values to the risk assessment [40]. In the present study, the application of serum dhS1P/dhSph ratio in early prediction of incident diabetes showed comparable accuracy to the conventional risk model before the changes of blood glucose. Combination of dhS1P levels and dhS1P/dhSph ratio with those conventional risk factors might provide a promising tool for early prediction of T2DM clinically.

The finding that dhS1P and dhS1P/dhSph ratio are elevated in serum up to 5 years before diabetes onset suggests that SphK2 activity could be stably associated with T2DM predisposition. Indeed, in recent years, an increasing body of evidence has suggested an important role of SphK2 in both insulin resistance and pancreatic β-cell dysfunction, the two key pathogenic factors of diabetes [10, 12, 41]. While SphK1 has been identified as protective factor against β-cell death [9], we have recently uncovered that SphK2 is an endogenous pro-apoptotic factor promoting β-cell lipotoxicity and dysfunction [10]. Global ablation of *Sphk2* significantly reserves β-cell mass and insulin production, resulting in amelioration of diabetic phenotype in a mouse model of T2DM [10]. Intriguingly, SphK2 is likely a metabolically protective factor in the liver, as *Sphk2*^−/−^ predisposes to non-alcoholic and alcoholic fatty liver diseases [42, 43], whereas adenoviral overexpression of SphK2 primarily in the liver improves insulin resistance [41]. More recently, we found that hepatocyte-specific *Sphk2*-deficent mice exhibited evident insulin resistance and pre-diabetes [12]. Thus, it appears that SphK2 can exert both protective and pathogenic effects, leading to different metabolic outcomes in a highly context-dependent manner. Although we are currently unable to assess the contribution of these different functions of SphK2 to the overall metabolic homeostasis, the association of circulating dhS1P/dhSph ratio with T2DM incidence has shed light on an important role of SphK2 in diabetes in a clinically relevant setting.

In this study, all cases and control subjects were strictly matched with respects to age, sex, BMI, FPG at baseline. Intriguingly, levels of total cholesterol in the incident diabetic group were higher than the control group. It is well acknowledged that total cholesterol is an established risk factor for diabetes. To exclude the potential confounding factor of cholesterol, we first performed a correlation analysis and found that dhS1P and dhS1P/dhSph ratio were not related to total cholesterol (*P* > 0.10). Moreover, in the logistic regression, after adjustment for total cholesterol, parameter estimates of the correlation between baseline dhS1P level and dhS1P/dhSph ratio and risk of incident diabetes remained significant. Hence, it is more likely to be a happenchance but not a real phenomenon associated with the changes in SphK-related sphingolipid metabolite levels.

It is interesting to note that high levels of serum dihydroceramides have recently been identified as a potential biomarker of T2DM susceptibility in both mice and humans [28]. Dihydroceramides, along with dhSph and dhSph, represent a key subspecies of sphingolipids, namely dihydrosphingolipids, which does not contain a C4-trans-double bond in the sphingoid backbone. They are produced in early steps of the de novo ceramide synthetic pathway (reviewed in [44]). Dihydroceramides are yielded from the conversion of dhSph by (dihydro-)ceramide synthases, and the latter can be phosphorylated by SphK2 to form dhS1P. Theoretically, loss of *Sphk2* leads to dhSph accumulation, which would increase dihydroceramide formation. Likewise, elevated SphK2 activity and increased dhS1P would result in a reduction of dihydroceramides. However, as dihydroceramides were not measured in the present study, we are unable to ascertain whether the individuals who had high levels of dhS1P with incident T2DM also had changes in dihydroceramides. This is worth to be tested in the future.

There are several limitations in the present study. First, while the nested case–control design has provided us a powerful tool to identify serum dhS1P/dhSph ratio as a biomarker for T2DM prediction in a given cohort, we did not evaluate its performance in additional large-scale population of cohorts due to funding constraints. Thus, the predictive value of serum dhS1P and the ratio of dhS1P/dhSph for incident T2DM in general population remains to be further validated. Second, our study participants were only of Han Chinese, and thus the further studies on a variety of races and ethnicities are required. Third, all participants in this study were middle-aged and elderly adults, and further work is needed to determine whether our findings can be extrapolated to all-ages population. Finally, in the present study we only measured circulating levels of the sphingolipid metabolites mostly related to SphK activity, including Sph, S1P, dhSph and dhS1P. Further work is needed to examine whether combining the current findings with more sphingolipid measurements, such as ceramides, dihydroceramides and glucosylceramides, could further improve the predictive value for incident T2DM.

In summary, we have shown that high levels of circulating dhS1P and its ratio to dhSph are prospectively associated with T2DM incidence in a prospective cohort, revealing a novel biomarker for susceptibility to develop diabetes, up to 5 years before the disease is diagnosed. Further studies are warranted to validate its prediction power in a general population and further understand the mechanisms underlying the effects of SphK-related sphingolipids in the pathogenesis of T2DM.

## Data Availability

The datasets during and/or analyzed during the current study are available from the corresponding author on reasonable request.

## References

[1] Saeedi P, Petersohn I, Salpea P, Malanda B, Karuranga S, Unwin N, Colagiuri S, Guariguata L, Motala AA, Ogurtsova K (2019). Global and regional diabetes prevalence estimates for 2019 and projections for 2030 and 2045: Results from the International Diabetes Federation Diabetes Atlas, 9(th) edition. Diabetes Res Clin Pract.

[2] Saeedi P, Salpea P, Karuranga S, Petersohn I, Malanda B, Gregg EW, Unwin N, Wild SH, Williams R (2020). Mortality attributable to diabetes in 20–79 years old adults, 2019 estimates: Results from the International Diabetes Federation Diabetes Atlas, 9(th) edition. Diabetes Res Clin Pract.

[3] Gregg EW, Cheng YJ, Srinivasan M, Lin J, Geiss LS, Albright AL, Imperatore G (2018). Trends in cause-specific mortality among adults with and without diagnosed diabetes in the USA: an epidemiological analysis of linked national survey and vital statistics data. Lancet (London, England).

[4] Chatterjee S, Khunti K, Davies MJ (2017). Type 2 diabetes. Lancet (London, England).

[5] Hannun YA, Obeid LM (2018). Sphingolipids and their metabolism in physiology and disease. Nat Rev Mol Cell Biol.

[6] Spiegel S, Milstien S (2003). Sphingosine-1-phosphate: an enigmatic signalling lipid. Nat Rev Mol Cell Biol.

[7] Pitson SM (2011). Regulation of sphingosine kinase and sphingolipid signaling. Trends Biochem Sci.

[8] Xia P, Wadham C (2011). Sphingosine 1-phosphate, a key mediator of the cytokine network: juxtacrine signaling. Cytokine Growth Factor Rev.

[9] Qi Y, Chen J, Lay A, Don A, Vadas M, Xia P (2013). Loss of sphingosine kinase 1 predisposes to the onset of diabetes via promoting pancreatic beta-cell death in diet-induced obese mice. Faseb J.

[10] Song Z, Wang W, Li N, Yan S, Rong K, Lan T, Xia P (2019). Sphingosine kinase 2 promotes lipotoxicity in pancreatic β-cells and the progression of diabetes. Faseb J.

[11] Anderson AK, Lambert JM, Montefusco DJ, Tran BN, Roddy P, Holland WL, Cowart LA (2020). Depletion of adipocyte sphingosine kinase 1 leads to cell hypertrophy, impaired lipolysis, and nonalcoholic fatty liver disease. J Lipid Res.

[12] Aji G, Huang Y, Ng ML, Wang W, Lan T, Li M, Li Y, Chen Q, Li R, Yan S (2020). Regulation of hepatic insulin signaling and glucose homeostasis by sphingosine kinase 2. Proc Natl Acad Sci U S A.

[13] Gao X, Hofman A, Hu Y, Lin H, Zhu C, Jeekel J, Jin X, Wang J, Gao J, Yin Y (2010). The Shanghai Changfeng Study: a community-based prospective cohort study of chronic diseases among middle-aged and elderly: objectives and design. Eur J Epidemiol.

[14] Xia MF, Lin HD, Chen LY, Wu L, Ma H, Li Q, Aleteng Q, Hu Y, He WY, Gao J (2019). The PNPLA3 rs738409 C>G variant interacts with changes in body weight over time to aggravate liver steatosis, but reduces the risk of incident type 2 diabetes. Diabetologia.

[15] Matthews DR, Hosker JP, Rudenski AS, Naylor BA, Treacher DF, Turner RC (1985). Homeostasis model assessment: insulin resistance and beta-cell function from fasting plasma glucose and insulin concentrations in man. Diabetologia.

[16] World Health Organization. Definition and diagnosis of diabetes mellitus and intermediate and hyperglycaemia. Report of a WHO/IDF consultation. http://www.who.int/diabetes/publications/diagnosis_diabetes2006/en/ (June 14 2019)

[17] Hammad SM, Pierce JS, Soodavar F, Smith KJ, Al Gadban MM, Rembiesa B, Klein RL, Hannun YA, Bielawski J, Bielawska A (2010). Blood sphingolipidomics in healthy humans: impact of sample collection methodology. J Lipid Res.

[18] Sullards MC, Liu Y, Chen Y, Merrill AH (2011). Analysis of mammalian sphingolipids by liquid chromatography tandem mass spectrometry (LC-MS/MS) and tissue imaging mass spectrometry (TIMS). Biochem Biophys Acta.

[19] Sensken SC, Bode C, Nagarajan M, Peest U, Pabst O, Gräler MH (2010). Redistribution of sphingosine 1-phosphate by sphingosine kinase 2 contributes to lymphopenia. J Immunol..

[20] Kharel Y, Huang T, Salamon A, Harris TE, Santos WL, Lynch KR (2020). Mechanism of sphingosine 1-phosphate clearance from blood. Biochem J.

[21] Hla T, Dannenberg AJ (2012). Sphingolipid signaling in metabolic disorders. Cell Metab.

[22] Chavez JA, Summers SA (2012). A ceramide-centric view of insulin resistance. Cell Metab.

[23] Lemaitre RN, Yu C, Hoofnagle A, Hari N, Jensen PN, Fretts AM, Umans JG, Howard BV, Sitlani CM, Siscovick DS (2018). Circulating Sphingolipids, Insulin, HOMA-IR, and HOMA-B: The Strong Heart Family Study. Diabetes.

[24] Chew WS, Torta F, Ji S, Choi H, Begum H, Sim X, Khoo CM, Khoo EYH, Ong WY, Van Dam RM (2019). Large-scale lipidomics identifies associations between plasma sphingolipids and T2DM incidence. JCI Insight.

[25] Huynh K, Barlow CK, Jayawardana KS, Weir JM, Mellett NA, Cinel M, Magliano DJ, Shaw JE, Drew BG, Meikle PJ (2019). High-throughput plasma lipidomics: detailed mapping of the associations with cardiometabolic risk factors. Cell Chem Biol.

[26] Levy M, Futerman AH (2010). Mammalian ceramide synthases. IUBMB Life.

[27] Petersen MC, Shulman GI (2017). Roles of diacylglycerols and ceramides in hepatic insulin resistance. Trends Pharmacol Sci.

[28] Wigger L, Cruciani-Guglielmacci C, Nicolas A, Denom J, Fernandez N, Fumeron F, Marques-Vidal P, Ktorza A, Kramer W, Schulte A (2017). Plasma dihydroceramides are diabetes susceptibility biomarker candidates in mice and humans. Cell Rep.

[29] Xia JY, Holland WL, Kusminski CM, Sun K, Sharma AX, Pearson MJ, Sifuentes AJ, McDonald JG, Gordillo R, Scherer PE (2015). Targeted induction of ceramide degradation leads to improved systemic metabolism and reduced hepatic steatosis. Cell Metab.

[30] Mizugishi K, Yamashita T, Olivera A, Miller GF, Spiegel S, Proia RL (2005). Essential role for sphingosine kinases in neural and vascular development. Mol Cell Biol.

[31] Pappu R, Schwab SR, Cornelissen I, Pereira JP, Regard JB, Xu Y, Camerer E, Zheng YW, Huang Y, Cyster JG (2007). Promotion of lymphocyte egress into blood and lymph by distinct sources of sphingosine-1-phosphate. Science.

[32] Olivera A, Allende ML, Proia RL (2013). Shaping the landscape: metabolic regulation of S1P gradients. Biochem Biophys Acta.

[33] Neubauer HA, Pitson SM (2013). Roles, regulation and inhibitors of sphingosine kinase 2. FEBS J.

[34] Venkataraman K, Thangada S, Michaud J, Oo ML, Ai Y, Lee YM, Wu M, Parikh NS, Khan F, Proia RL (2006). Extracellular export of sphingosine kinase-1a contributes to the vascular S1P gradient. Biochem J.

[35] Merrill AH, Sullards MC (2017). Opinion article on lipidomics: inherent challenges of lipidomic analysis of sphingolipids. Biochim Biophys Acta.

[36] Chong JR, Xiang P, Wang W, Hind T, Chew WS, Ong WY, Lai MKP, Herr DR (2018). Sphingolipidomics analysis of large clinical cohorts. Part 2: Potential impact and applications. Biochem Biophys Res Commun.

[37] Wang TJ, Larson MG, Vasan RS, Cheng S, Rhee EP, McCabe E, Lewis GD, Fox CS, Jacques PF, Fernandez C (2011). Metabolite profiles and the risk of developing diabetes. Nat Med.

[38] Chen C, Chen Q, Nie B, Zhang H, Zhai H, Zhao L, Xia P, Lu Y, Wang N (2020). Trends in bone mineral density, osteoporosis, and osteopenia among U.S. adults with prediabetes, 2005–2014. Diabetes Care.

[39] Kelsey MM, Pyle L, Hilkin A, Severn CD, Utzschneider K, Van Pelt RE, Nadeau KJ, Zeitler PS (2020). The impact of obesity on insulin sensitivity and secretion during pubertal progression: a longitudinal study. J Clin Endocrinol Metab.

[40] Meigs JB, Shrader P, Sullivan LM, McAteer JB, Fox CS, Dupuis J, Manning AK, Florez JC, Wilson PW, D'Agostino RB (2008). Genotype score in addition to common risk factors for prediction of type 2 diabetes. N Engl J Med.

[41] Lee SY, Hong IK, Kim BR, Shim SM, Sung Lee J, Lee HY, Soo Choi C, Kim BK, Park TS (2015). Activation of sphingosine kinase 2 by endoplasmic reticulum stress ameliorates hepatic steatosis and insulin resistance in mice. Hepatology (Baltimore, MD).

[42] Kwong EK, Liu R, Zhao D, Li X, Zhu W, Wang X, Gurley EC, Lai G, Liu J, Hylemon PB (2019). The role of sphingosine kinase 2 in alcoholic liver disease. Digestive Liver Dis.

[43] Nagahashi M, Takabe K, Liu R, Peng K, Wang X, Wang Y, Hait NC, Wang X, Allegood JC, Yamada A (2015). Conjugated bile acid-activated S1P receptor 2 is a key regulator of sphingosine kinase 2 and hepatic gene expression. Hepatology (Baltimore, MD).

[44] Magaye RR, Savira F, Hua Y, Kelly DJ, Reid C, Flynn B, Liew D, Wang BH (2019). The role of dihydrosphingolipids in disease. Cell Mol Life Sci.

